# Frogs with denser group-spawning mature later and live longer

**DOI:** 10.1038/s41598-019-50368-w

**Published:** 2019-09-24

**Authors:** Yun Lin Cai, Chun Lan Mai, Wen Bo Liao

**Affiliations:** 1 Department of Urology, The Second Clinical Medical College of North Sichuan Medical College, Nanchong, 637000 China; 2 Key Laboratory of Southwest China Wildlife Resources Conservation (Ministry of Education), China West Normal University, Nanchong, 637009 Sichuan China; 3 Key Laboratory of Artificial Propagation and Utilization in Anurans of Nanchong City, China West Normal University, Nanchong, Sichuan 637009 China; 4 Institute of Eco-adaptation in Amphibians and Reptiles, China West Normal University, Nanchong, 637009 Sichuan China

**Keywords:** Evolutionary ecology, Evolutionary developmental biology

## Abstract

The understanding of the intrinsic and extrinsic causes of longevity variation has deservedly received much attention in evolutionary ecologist. Here we tested the association between longevity and spawning-site groups across 38 species of Chinese anurans. As indicators of group-spawning we used spawning-site group size and spawning-site density, which we measured at 152 spawning sites in the field. We found that both spawning-site density and group size were positively associated with longevity. Male group-spawning (e.g., male spawning-site density and male spawning-site group size) was also positively correlated with longevity. A phylogenetic path analysis further revealed that longevity seems directly associated with spawning-site density and group size, and that the association in part depend on the ‘groups-spawning-age at first reproduction’ association. Our findings suggest that the increased group-spawning are likely to benefit in declining extrinsic mortality rates and living longer through improving total anti-predator behaviour under predation pressure.

## Introduction

Longevity varies greatly both within and among species and populations in animals^1–9^ and understanding the proximate and ultimate causes of this variation has deservedly received much attention in evolutionary biologists^10–18^. The proximate causes are the results of hazards from the environments, such as predator risk, diseases, parasitism, famine, competition, drought or accidents^19,20^. Meanwhile, the ultimate causes result from intrinsic processes of physical and functional degradation originating within the body, such as spontaneous chemical reactions, replication errors and accumulation of metabolic waste products^20^.

Senescence can be explained by a decline in the power of natural selection with age^21,22^. The senescence theory states that members of populations exposed to lower degree of extrinsic mortality will evolve longer potential longevity^2,17^. In particular, animals suffering lower extrinsic mortality rates can postpone age at first reproduction, placing organisms under additional stress, which can, in turn, decrease intrinsic mortality^23^. This can select for a longer longevity so that potential reproduction is maximized^2^. From those preceding studies, it is evident that age at first reproduction is important predictor of longevity^24^. For instance, maximum longevity is positively correlated with age at first reproduction in taxa^14,16,25–27^.

Most studies have found longevity to be strongly associated with both intrinsic and extrinsic mortality rates in animal kingdoms^2,4,17,28–31^. For example, the use of poison and nocturnality, which ascribe to reduced predation pressure (low extrinsic mortality rate) are associated with longer longevity in amphibians^4^. Stark *et al*. found reptilian, that live on islands, and in colder and more seasonal environments, presumably suffer lower rates of predation, live longer^18^. Additionally, group size is an important life-history trait that is expected to affect longevity^12,26^. Species living in larger groups should exhibit lower extrinsic mortality rate because aggregations in animals typically decrease *per capita* predation risk^32,33^, and thereby possessing longer longevity. However, there are evidences that longevity is not correlated with group-foraging and sociality in North American birds^14,15^ and group size in mammals^27,31^. For anurans, group spawners typically aggregate in ponds where the density of individuals varies across species^34^. The measure can be used as proxy for extrinsic mortality rate where denser aggregations indicate lower predation risk because denser or larger aggregations have been shown to (i) yield survival benefits to individuals, (ii) to increase total anti-predator behaviour, and (iii) to reduce the costs of anti-predator behaviours^35–42^. In addition, competition is the other force that drives the evolution of life history^43^. According to life history theory, increasing population size would enhance competition, the K-selected strategy would be favored in this scenario that eventually shapes the whole life history, such as prolonged longevity^12^.Although the factors (e.g., chemical protection, activity period, microhabitat preferences and annual temperature) influencing longevity have been tested formally in amphibians^4,29,44,45^, the relationship between group-spawning and longevity in anurans remains untested.

Here, we evaluated the association between longevity and group-spawning (e.g., spawning-site density and spawning-site group size) among 38 species of anurans when controlling the associated variables (e.g., altitude, latitude, age at first reproduction and SVL: snout-vent length) which may affect longevity variation. Larger group-spawners have lower predation pressures, which result in lower extrinsic mortality rate^12^. Hence, we predict that frogs with denser group-spawning should mature later and live longer.

## Results

Group-spawning (e.g., spawning-site group density and spawning-site group size) tended to be correlated with SVL in our sample of 38 frog species (Table S1). Group-spawning was further closely correlated with age at first reproduction (Table S1). PGLS revealed that longevity was positively correlated with age at first reproduction and SVL (*P* < 0.001 and *P* = 0.022, respectively), and tended to be positively correlated with altitude and latitude, respectively (*P* = 0.093 and *P* = 0.057; Table S2).

**Figure 1:**
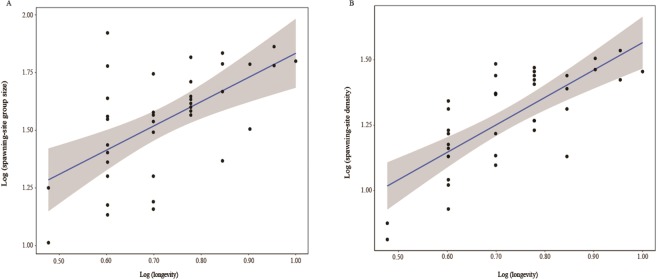
Relationships between group-spawning (e.g., spawning-site density and spawning-site group size) and longevity across 38 species of frogs. The shaded areas indicated 95% confidence interval and the blue line indicated significant correlation between group-spawning and longevity.

**Figure 2:**
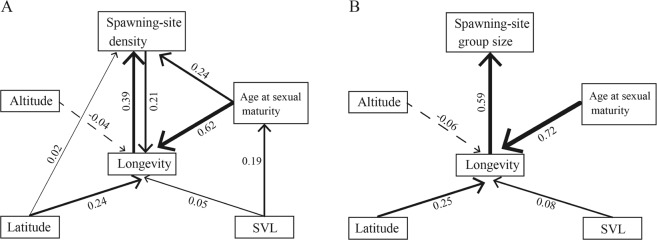
Visual representation of the averaged best-fitting path models (ΔCICc ≤ 2) for the anurans. Arrows reflect the direction of the path, and their line width is proportional to their standardized regression coefficients (adjacent to arrows).

Longevity was positively correlated with group-spawning (Fig. 1; spawning-site density: *β* = 0.52, *t* = 6.54, *P* < 0.001, λ < 0.001^1, <0.001^; spawning-site group size: *β* = 0.33, *t* = 4.34, *P* < 0.001, λ < 0.001^1, <0.001^). Longevity sample size did not affect this relationship between spawning-site density and longevity (*β* = −0.002, *t* = −0.233, *P* = 0.818, λ < 0.001^1, <0.001^) and between longevity and spawning-site group size (*β* = 0.005, *t* = 0.453, *P* = 0.654, λ < 0.001^1, <0.001^). Group-spawning was positively correlated with longevity when controlling the effects of age at first reproduction, altitude, latitude and SVL (Table 1). The association between group-spawning and longevity was dependent on the ‘spawning groups-age at first reproduction’ association which was supported by our phylogenetic confirmatory path analyses (Figs 2 and S1, S2; Tables S3, S4). When using mean age (average age of all individuals for every species), instead of maximum lifespan as proxy for longevity and relating it to group-spawning, our results remained qualitatively similar as frogs with denser group-spawning were older (Table 1).

**Table 1:** PGLS model of relationships between lifespan/mean age and group-spawning for 38 species of anurans.

Predictors	Longevity				Mean age			
	*λ*	*β*	*t*	*P*	*λ*	*β*	*t*	*P*
Spawning-site density	<0.001^1,<0.001^	0.308	4.385	<0.001	<0.001^1,0.002^	0.213	2.551	0.016
SVL		0.040	0.498	0.622		0.201	2.100	0.044
Altitude		−0.008	−0.235	0.816		0.070	1.711	0.097
Latitude		1.076	2.587	0.014		1.218	2.453	0.020
Age at sexual maturity		0.350	4.265	<0.001		0.284	2.879	0.007
Spawning-site group size	<0.001^1,<0.001^	0.167	2.827	0.008	<0.001^1,0.012^	0.115	1.795	0.082
SVL		0.053	0.580	0.566		0.209	2.089	0.045
Altitude		−0.011	−0.290	0.774		0.071	1.658	0.107
Latitude		1.130	2.401	0.022		1.266	2.448	0.020
Age at sexual maturity		0.424	4.790	<0.001		0.337	3.445	0.002

Significant predictors are marked in bold. Phylogenetic scaling parameters (superscripts following λ denote *P*-values of likelihood ratio tests against models with λ = 0 and λ = 1, respectively).

Longevity was positively correlated with male group-spawning (male spawning-site density: *β* = 0.44, *t* = 6.14, *P* < 0.001, λ < 0.001^1, <0.001^; male spawning-site group size: *β* = 0.39, *t* = 5.78, *P* < 0.001, λ < 0.001^1, <0.001^). Longevity and mean age were positively correlated with male group-spawning when controlling the effects of age at first reproduction, altitude, latitude and SVL (Table S5). Hence, when using male group-spawning instead of group-spawning, we found a same result.

## Discussion

We first time investigated the relationship between group-spawning and longevity outside of homeothermic taxa. We found a positive association between longevity and group-spawning after controlling for several potential confounding factors and phylogenetic non-independence, which is inconsistent with what has previously been shown in birds^14,15^ and mammals^27^. Spawning aggregations of anurans with longer longevity were denser than spawning aggregations with shorter longevity. Our findings suggest that evolving a longer longevity is beneficial when denser group-spawning may result in lower extrinsic mortality by declining predation risk^36,37^. Below we discuss what may underlie this consistent pattern of longevity variation across vertebrate taxa.

Selection for larger body size leads to better predator avoidance^46^. Larger-bodied species experience fewer predators than smaller-bodied species, thereby reducing mortality risk^47–49^. In the case where larger-bodied species can increase survival, thereby living longer. Alternatively, larger-bodied species have lower basal metabolic rate which is correlated with increased longevity in endotherms^50^. In particular, there are evidences that body size is positively correlated with longevity across taxa in both endotherms and ectotherms ^2,4,5,27,30,51^. Body size is positively associated with longevity across amphibians and within the large amphibian orders^4^. The trade-off between growth and reproduction can the general pattern because the growth of a large size need take longer development time and delay reproduction, and this selects for a longer longevity^31^. We found that longevity was positively correlated with SVL across 38 species of anurans, similar with previous studies on amphibians^30,51–54^.

Most studies have shown that longevity is positively correlated with age at first reproduction in anurans^1,25,55^. This positive correlation can be explained by life-history trade-off^12^ that growing to larger body and longer longevity need delays reproduction, thus selecting for prolonged age at first reproduction. Path analyses also suggested the positive relationship between group-spawning and longevity depending on the positive relationship between group-pawning and age at first reproduction. Moreover, we found that longevity sample size did not affect relationship between longevity and group-spawning. It appears that increasing sample size does not strongly increase the probability of finding older individuals in our study.

Previous studies have shown the different relationships between social groups (e.g., group-foraging, complex sociality, and colony size) and longevity^29,56–58^). In comparative studies, species living in larger social groups do not display longer longevity when controlling for other factors known to affect longevity in birds^14,15,29,56^ and in mammals^27,31^. However, social breeding was positively linked to increased longevity and survival rates in birds when phylogenetic effect and sampling effort was not controlled^57,58^. The findings result from the argument that species living in social groups confers benefits to group members, consequently reducing mortality rates and increasing longevity^12,59^. Our anurans results showed a positive effect of spawning-group on longevity, suggesting that frogs with denser group-spawning faced to lower predation risk, and thereby decreasing mortality rates and living longer. By contrast, Kamilar *et al*. found a weak and negative correlation between group size and longevity in artiodactyls^27^. The shorter longevity may result from higher rates of extrinsic mortality in larger groups where they are more conspicuous to predators in open habitats^27^.

The cognitive buffer hypothesis (CBH) predicts that the increased cognitive abilities provided by a larger brain can facilitate appropriate behavioral responses toward uncommon or complex socioecological alterations^60–63^. The role of sexual selection^64^, especially mate choice^65^ in brain evolution has recently been addressed. For instance, humans evolved such large brains because the increased cognitive abilities associated with large brains are attractive to females^66^. For males, relatively larger brains and better cognitive abilities can likewise result in better male mate competition in the guppy^67^. According to life history theory, as population size or density increases, males will encounter comparatively more other males, and thus increasing intensity of competition for mating opportunities^68,69^. In this case, population size or density eventually shapes the whole life history based on the K-selected strategy^12^. This was also candidate causation to the positive association between spawning-site group size and longevity in this study. As a result, larger-brained anurans with better cognitive abilities were expected to experience denser spawning aggregations and stronger male mate competition than smaller-brained species. Previous studies have suggested that a larger brain facilitate the evolution of a longer lifespan via increasing individual survival probability in unpredictable situations^70,71^. Likewise, Yu *et al*. revealed that larger-brained anurans live longer in the wild^25^ because the developmental costs hypothesis predicts that a larger brain occurs as a by-product of a generally slower life history such as longer lifespan^72^. Hence, anurans displayed a positive association between spawning aggregations and longevity based on cognitive abilities and developmental costs.

Additionally, larger groups are shown to associate with higher levels of parasite infection^73^. However, there is no association between parasite species richness and host longevity in a large sample of anthropoid primates^74^ while a negative association is observed for 23 mammal species^75^. Additional studies are therefore needed to investigate the effect of parasite infection in different group size on longevity in anurans.

In conclusion, we found that frogs with denser spawning groups mature later and live longer and interpret this as the evidences for group-spawning increase resulting in decrease in mortality through improving anti-predator behaviour and cognitive adaptations to predation pressure^35–42^.

## Materials and Methods

### Ethical approval

All experimental methods were carried out in accordance with the current laws of China concerning animal experimentation, and permission to collect amphibians was received from the ethical committee for animal experiments in China Council on Animal Care (CCAC) guidelines. All experimental protocols were approved by the Animal Ethics Committee (AEC) at China West Normal University.

### Data collection

Spawning-site group size and density for 38 species was determined in breeding season (April to June) in the field between 2008 and 2017 according to the following procedure. Firstly, we located breeding ponds (four per species) and captured all individuals at each pond at night using a 12-V flashlight. The number of individuals was then counted and their sexes were determined by their secondary sexual characteristics (e.g., nuptial pad in males and the eggs readily visible through the skin of the abdomen in adult females). A red string was used to leg-marked individuals where they were released in their respective breeding ponds. The next day all individuals (including previously marked individuals) were recaptured, and we counted their numbers. All new individuals were leg-marked using yellow string and released in the pond. The third day, we again captured all individuals. We estimated the group size of each pond based on average number of individuals on the three days. In field, we selected all ponds where the shape did not vary and was always near rectangular to collect individuals. We then measured greatest length and width of pond to determine pond sizes and calculate surface area: A = length * width. Spawning-site group size was the number of individuals per pond and we calculated spawning-site density as the ratio between present individuals and the area of pond. For every species we determined average spawning-site group size, spawning-site density based on data on three days of all four ponds. Moreover, we estimated the male group size and density of each species. The estimated group-spawning size may change with the weather condition (e.g., raining or sunny day), and we collected group size of all species at raining day in their breeding peak. This case can control the effects of weather condition on the group-size data collection among different species. We also collected breeding season length of each species based on four spawning sites^38^.

### Age determination

Longevity data were the maximum lifespan (years) reported for each species. We obtained age at sexual maturity, mean age, and longevity for 38 species from published literature^25^ (based on skeletochronology, Table S6). Because the estimate of longevity could be based on unequal numbers of individuals for different species, sample size may affect estimate of maximum lifespan. In this study, with an increasing number of sampled individuals the likelihood of sampling a particularly old individual should increase. As sample size per species ranged between 20 and 141 individuals we corrected for some of the potential biases inherent in the use of maxima resulting from the longevity sample size when longevity was estimated for each species. In particular, we treated longevity as the response variable, group-spawning as the predictor variable, and longevity sampling size as a covariate to correct the effect of sampling size on the relationship between longevity and group-spawning.

### Associated variables

Other traits may co-vary with longevity and group-spawning. It is therefore important to investigate their relationship with group-spawning and longevity due to the correlative nature of comparative analyses. Covariation of life-history traits is common across species^76,77^, we therefore extracted information on life-history traits (e.g., breeding season length and age at sexual maturity) that are typically correlated with longevity from our own previous studies^25^ (Table S6). Moreover, environmental factors (mean annual temperatures and rainfall) affect longevity variation^78^. We collected everyday average temperature and rainfall at each location and calculated mean annual temperatures and rainfall from Chinese Meteorological Stations (http://www.lishi.tianqi.com) between 2011 and 2015^79^. Also, longevity can be affected by geography^44,80^. For instance, latitude is a strong predictor for longevity in birds^81^ while altitude is positively correlated with longevity in anurans^44^. We therefore included altitude and latitude in our analyses (see below).

### Phylogeny

We reconstructed a molecular phylogeny with a full coverage of 38 species following the methods of the reference^25^ (Fig. S3). We obtained the GenBank accession numbers (Table S7). BEAUTi and BEAST v.1.8.3^82^ with unlinked substitution models, a relaxed uncorrelated lognormal clock, a Yule speciation process, and no calibration points due to a lack of fossil dates, were used to constructed phylogenies (Fig. S3). We made the Markov Chain Monte Carlo (MCMC) simulation to run for 100 million generations and sampled a tree every 5000^th^ generation. The satisfying convergence of the Bayesian chain and adequate model mixing for each of the tree statistics was shown the effective sample size (ESS) in the program Tracer v.1.6.0^83^. We used TreeAnmtator V.1.8.3^82^ to generate a maximum clade credibility tree with mean node heights and a 10% burn-in.

### Data analyses

All statistical analyses were conducted in the R statistical environment version 3.3.1^84^, and all data was log_10_-transformed to meet normal distribution. We accounted for the phylogenetic structure of the model residuals using phylogenetic generalized least-squares (PGLS) models in the R package *caper*^85^ and our reconstructed phylogeny. Using a maximum-likelihood approach, we evaluated the phylogenetic effect on relationships through phylogenetic scaling parameter λ^86,87^. These λ values cover a scale raging from 0 (phylogenetic nonindependence) to 1^88,89^ (complete phylogenetic dependence). To investigate the relationships between group-spawning, environmental factors (e.g., altitude, latitude, temperature and rainfall) and life-history traits (breeding season length, age at sexual maturity and body size) we first used a PGLS models treating group-spawning as the response variable, and environmental factors or life-history traits as the predictor variable. To then investigate the factors of longevity variation, we examined the relationships between longevity and environmental factors (e.g., altitude, latitude, temperature and rainfall) and life-history traits in another PGLS approach, treating longevity as the response variable, environmental factors or life-history traits as predictor variables. Finally, for the analysis of the relationship between group-spawning and longevity we used the PGLS models treating longevity as the response variable, group-spawning as the predictor variable, and age at first reproduction, altitude, latitude and SVL as covariates.

Longevity and group-spawning may be directly linked; they may also be indirectly associated via changes in age at first reproduction, altitude, latitude and SVL. Hence, phylogenetic confirmatory path analyses^90^ on the basis of pre-specified candidate path models in the R package *phylopath* v1.0.0^91^ were performed to test the associations between longevity, age at first reproduction, altitude, latitude and SVL and group-spawning. To evaluate the models, we compared a total of 20 models with different configurations of these variables (Supplementary Information: Figs S1, S2) using the C-statistic Information Criterion (CICc) corrected for small sample sizes. A path analysis approach in the R package phylopath v1.0.0^91^ ranked all candidate models based on their C-statistic Information Criterion (CICc) and averaged those with ΔCICc ≤ 2 from the top model was used to examine the conditional independences of each model. Path coefficients are averaged only over models where the path exists^90^.

## Supplementary information

Supplementary
