# Supplementary material for: Frogs with denser group-spawning mature later and live longer
Source: Sci Rep. 2019 Sep 24;9:13776. doi: 10.1038/s41598-019-50368-w (PMC6760165; doi:10.1038/s41598-019-50368-w)

Electronic Supplementary Material

Frogs with denser group-spawning mature later and live longer

Yun Lin Cai[1#], Chun Lan Mai[2#], Wen Bo Liao[2,3,4]

[1]Department of Urology, the Second Clinical Medical College of North Sichuan Medical College, Nanchong, 637000, China

[2]Key Laboratory of Southwest China Wildlife Resources Conservation (Ministry of Education), China West Normal University, Nanchong, 637009, Sichuan, China

[3]Key Laboratory of Artificial Propagation and Utilization in Anurans of Nanchong City, China West Normal University, Nanchong, Sichuan, 637009, China

[4]Institute of Eco-adaptation in Amphibians and Reptiles, China West Normal University, Nanchong, 637009, Sichuan, China

# These authors contributed equally.

*Corresponding author: Wen Bo Liao, E-mail: liaobo_0_0@126.com

Table S1 Relationships between group-spawning (e.g., spawning-site density and spawning-site group size) and altitude, latitude, rainfall, temperature, breeding season length, age at sexual maturity, and body size in anurans using a PGLS model.

Predictors	Spawning-site density				Spawning-site group size			
	λ	*β*	*t*	*P*	*λ*	*β*	*t*	*P*
SVL	$<0.001^{1,<0.001}$	0.359	1.827	0.076	$<0.001^{1,<0.001}$	0.370	1.472	0.150
Altitude	$<0.001^{1,<0.001}$	0.073	0.859	0.396	$<0.001^{1,<0.001}$	0.057	0.524	0.603
Latitude	$<0.001^{1,<0.001}$	0.840	0.730	0.470	$<0.001^{1,<0.001}$	0.996	0.686	0.497
Rainfall	$<0.001^{1,<0.001}$	0.014	0.068	0.946	$<0.001^{1,<0.001}$	-0.424	-1.774	0.085
Temperature	$<0.001^{1,<0.001}$	-0.065	-0.971	0.338	$<0.001^{1,<0.001}$	-0.076	-0.897	0.376
Breeding season length	$<0.001^{1,<0.001}$	-0.300	-2.662	0.012	$<0.001^{1,<0.001}$	-0.116	-0.753	0.456
Age at sexual maturity	**$<0.001^{1,<0.001}$**	**0.533**	**3.733**	**<0.001**	**$<0.001^{1,<0.001}$**	**0.528**	**2.746**	**0.009**

Table S2 Relationships between longevity and altitude, latitude, rainfall, temperature, breeding season length, age at sexual maturity, sample size and body size in anurans using a PGLS model. Phylogenetic scaling parameters (superscripts following λ denote *P*-values of likelihood ratio tests against models with λ = 0 and λ = 1, respectively).

Predictors	Longevity			
	λ	β	t	P
SVL	$<0.001^{1,<0.001}$	0.322	2.399	0.022
Altitude	$0.350^{0.296,<0.001}$	0.095	1.724	0.093
Latitude	$<0.001^{1,<0.001}$	1.522	1.963	0.057
Rainfall	$0.327^{0.607,<0.001}$	-0.058	-0.429	0.671
Temperature	$0.322^{0.432,<0.001}$	-0.081	-1.905	0.065
Sample size	$<0.001^{1,<0.001}$	0.202	1.843	0.074
Breeding season length	$<0.001^{1,<0.001}$	-0.234	-3.027	0.005
Age at sexual maturity	$<0.001^{1,<0.001}$	0.533	6.841	<0.001

Table S3: Results of association between longevity and spawning-site density through age at sexual maturity using the phylogenetic path analyses, ranking the candidate models (visual representation in Fig. S1) according to their CICc. The models with ΔCICc < 2 are represented in bold and were used to calculate the average model (Fig. 2).

**Model**	***k***	***q***	***C***	***CICc***	***ΔCICc***	***W_i***
m17	**10**	**11**	**31.863**	**0.045**	**64.017**	**<0.001**
m13	**8**	**13**	**24.409**	**0.081**	**65.576**	**1.559**
m4	**8**	**13**	**24.632**	**0.077**	**65.798**	**1.782**
m5	9	12	30.369	0.034	66.849	2.832
m15	9	12	31.500	0.025	67.980	3.963
m1	9	12	31.596	0.025	68.076	4.060
m3	9	12	31.936	0.022	68.416	4.399
m8	7	14	23.460	0.053	69.721	5.704
m14	8	13	28.752	0.026	69.919	5.902
m9	8	13	30.987	0.014	72.153	8.137
m20	8	13	31.031	0.013	72.197	8.180
m19	9	12	37.216	0.005	73.696	9.680
m7	10	11	41.906	0.003	74.060	10.043
m11	9	12	38.649	0.003	75.129	11.112
m6	9	12	38.904	0.003	75.384	11.367
m18	8	13	35.757	0.003	76.924	12.907
m2	10	11	49.009	<0.001	81.163	17.146
m10	10	11	49.695	<0.001	81.849	17.832
m12	9	12	46.756	<0.001	83.236	19.219
m16	10	11	57.025	<0.001	89.178	25.162

K = number of independence claims; q = number of parameters; C = Fisher’s C statistics; CICc = C-statistic Information Criterion; ΔCICc, difference in CICc from the best-fitting model; wi, CICc weights.

Table S4: Results of association between longevity and spawning-site group size through age at sexual maturity using the phylogenetic path analyses, ranking the candidate models (visual representation in Fig. S2) according to their CICc. The models with ΔCICc < 2 are represented in bold and were used to calculate the average model (Fig. 2).

**Model**	***k***	***q***	***C***	***CICc***	***ΔCICc***	***W_i***
m17	**10**	**11**	**30.606**	**0.061**	**62.760**	**<0.001**
m4	8	13	24.378	0.082	65.545	2.784
m13	8	13	24.408	0.081	65.574	2.814
m5	9	12	30.215	0.035	66.695	3.935
m15	9	12	30.260	0.035	66.740	3.979
m7	10	11	35.533	0.017	67.687	4.926
m1	9	12	31.343	0.026	67.823	5.063
m3	9	12	31.865	0.023	68.345	5.585
m8	7	14	23.270	0.056	69.531	6.771
m14	8	13	28.982	0.024	70.149	7.389
m20	8	13	29.580	0.020	70.747	7.986
m9	8	13	30.728	0.015	71.895	9.134
m10	10	11	40.990	0.004	73.144	10.384
m19	9	12	36.891	0.005	73.371	10.611
m11	9	12	37.647	0.004	74.127	11.367
m6	9	12	37.837	0.004	74.317	11.557
m18	8	13	35.987	0.003	77.154	14.394
m2	10	11	51.775	<0.001	83.929	21.169
m12	9	12	50.512	<0.001	86.992	24.232
m16	10	11	55.784	<0.001	87.938	25.178

K = number of independence claims; q = number of parameters; C = Fisher's C statistics; CICc = C-statistic Information Criterion; ΔCICc, difference in CICc from the best-fitting model; w_i, CICc weights.

Table S5. PGLS model of the relationships between lifespan/mean age and male group-spawning for 38 species of anurans. Significant predictors are marked in bold. Phylogenetic scaling parameters (superscripts following λ denote *P*-values of likelihood ratio tests against models with λ = 0 and λ = 1, respectively).

Predictors	Longevity				Mean age			
	λ	*β*	*t*	*P*	*λ*	*β*	*t*	*P*
Male spawning-site density	$<0.001^{1,<0.001}$	**0.265**	**4.306**	**<0.001**	$<0.001^{1,<0.001}$	**0.212**	**2.588**	**0.014**
SVL		0.038	0.463	0.646		**0.200**	**2.127**	**0.041**
Altitude		-0.006	-0.173	0.864		0.070	1.776	0.085
Latitude		**1.082**	**2.586**	**0.015**		**1.231**	**2.549**	**0.016**
Age at sexual maturity		**0.364**	**4.478**	**<0.001**		**0.288**	**3.019**	**0.005**
Male spawning-site group size	$<0.001^{1,<0.001}$	**0.229**	**3.933**	**<0.001**	$0.014^{0.970,0.004}$	0.115	1.819	0.078
SVL		-0.010	-0.119	0.906		**0.209**	**2.131**	**0.041**
Altitude		0.001	0.019	0.985		0.070	1.674	0.104
Latitude		**0.923**	**2.106**	**0.043**		**1.260**	**2.498**	**0.018**
Age at sexual maturity		**0.389**	**4.721**	**<0.001**		**0.336**	**3.534**	**0.001**

Table S6. Species, latitude (°), altitude (m), SVL (mm), longevity sample size, breeding season length (days), rainfall (mm), temperature (℃), spawning-site density (individuals/10m^2), spawning-site group size, age at sexual maturity (years) and longevity (mean age, years).

Species	Latitude	Altitude	SVL	Longevity sample size	Breeding season length	Rainfall	Temperature	Spawning-site density	Male Spawning-site density	Spawning-site group size	Male spawning-site group size	Age at sexual maturity	Longevity (mean age)
Amolops chunganensis	32.65	2450	36.5	25	19.0	563	7.82	29.0	17.27	61.1	36.39	2	8 (4.84)
Amolops lifanensis	30.88	2650	54.2	20	28.5	932	8.04	24.5	14.81	68.3	41.26	3	7 (3.8)
Amolops mantzorum	30.55	1700	52.35	76	58.5	1080	11.63	13.5	7.57	23.3	13.04	2	7(4.3)
Babina pleuraden	27.88	1413	54.5	24	45.3	551	16.83	11.0	5.58	27.3	13.82	2	4(2.7)
Bombina maxima	27.70	2685	49.0	40	27.5	861	10.33	28.5	17.44	65.5	40.09	2	6(3.5)
Brachytarsophrys chuannanensis	27.77	1860	102.2	25	22.0	1463	16.88	27.5	14.49	38.3	20.18	3	6(3.44)
Bufo andrewsi	30.55	1700	73.2	54	78.0	1080	11.63	27.5	18.35	61.3	40.87	2	7(3.8)
Bufo gargarizans	30.80	290	94.7	68	27.3	525	19.21	17.0	11.36	39.8	26.56	2	6(3.4)
Bufo melanostictus	27.38	1045	75.9	27	125.5	615	11.5	23.5	13.75	55.5	32.47	2	5(2.6)
Bufo minshanicus	32.05	1745	57.7	28	31.3	563	10.7	26.5	14.25	72.8	39.12	3	9(3.96)
Bufo tibetanus	30.52	2865	62.5	25	45.5	1123	0.32	23.3	14.54	31.0	19.34	2	5(3.12)
Feirana quadranus	32.30	1400	82.0	26	38.8	1160	11.63	15.0	8.37	43.5	24.27	1	4(3.8)
Fejervarya limnocharis	28.62	265	40.2	22	166.8	582	19.33	13.5	8.07	60.0	35.87	1	4(1.6)
Hyla annectans chuanxiensis	30.35	1413	33.0	33	43.8	885	15.5	10.5	5.63	13.6	7.31	1	4(2.1)
Hyla annectans jingdongensis	27.53	2028	35.3	26	51.0	1071	6.67	14.5	8.07	35.3	19.62	1	4 (2.3)
Hyla tsinlingensis	29.10	1749	40.5	27	46.0	1510	9.96	6.5	3.44	10.3	5.42	1	3 (1.6)
Hylarana guentheri	30.83	338	71.3	45	50.3	551	18.75	8.5	4.70	23.0	12.72	1	4(2.4)
Kaloula rugifera	32.30	1400	38.9	25	47.8	1166	11.63	17.0	10.37	15	9.15	1	4(1.92)

Kaloula verrucosa	27.47	1834	42.1	25	80.8	1016	14.75	22.0	14.34	83.5	11.57	1	4(2.28)
Microhyla ornata	30.83	320	22.4	25	164.8	686	19.25	7.5	3.63	17.8	8.59	1	3(1.56)
Nanorana parkeri	30.52	3210	45.6	39	47.0	616	0.43	28.5	15.88	63.0	35.10	3	10(3.5)
Nanorana ventripunctata	27.55	2028	44.1	25	107.5	1071	6.67	13.5	8.13	25.3	15.23	2	4(2.44)
Odorrana grahami	27.27	1860	75.6	40	24.3	885	12.37	26.5	17.50	43.0	28.40	2	6(3.7)
Odorrana margaretae	32.67	1046	81.4	25	30.3	1424	14.25	18.5	11.51	51.3	31.89	2	6(3.84)
Odorrana nanjiangensis	32.58	1468	55.6	27	27.0	1160	12.23	34.3	20.99	60.3	36.90	2	9(4.78)
Paa boulengeri	30.88	2650	98.67	25	101.3	932	6.29	16.5	7.67	36.8	17.08	2	5(2.72)
Paa yunnanensis	28.65	1350	86.95	25	79.0	1281	14.04	13.6	7.77	14.4	22.86	1	5(2.76)
Pelophylax hubeiensis	30.97	268	43.1	27	74.0	858	18.04	23.5	14.27	34.5	20.95	1	5(2.85)
Pelophylax nigromaculata	30.83	338	62.3	32	24.0	551	18.54	25.5	14.82	44.3	25.72	2	6 (2.4)
Polypedates megacephalus	29.83	668	44.5	27	44.8	708	16.79	27.5	16.30	20.0	11.85	2	5(2.9)
Rana chaochiaoensis	27.17	1935	53.6	25	32.3	885	11.5	16.5	8.69	20.0	10.53	1	4(2.08)
Rana chensinensis	32.60	546	46.9	63	19.3	1120	16.8	30.5	19.15	37.8	23.70	1	5(2.2,)
Rana kukunoris	31.50	3543	56.3	63	56.3	1088	-2.08	20.5	12.15	46.5	27.56	3	7(4.2)
Rana omeimontis	28.78	281	60.1	63	26.8	378	19.13	18.5	10.98	41.3	24.48	1	6(2.1)
Rhacophorus chenfui	28.30	380	36.3	26	64.5	1023	19.08	20.5	11.58	36.3	20.48	1	4(2.39)
Rhacophorus dennysi	27.77	456	81.3	26	36.8	1463	17.04	32.0	19.97	32.0	19.97	3	8 (4.35)
Rhacophorus dugritei	28.92	2548	43.8	26	24.5	1524	9.5	12.5	7.23	15.5	8.97	2	5 (2.92)
Rhacophorus omeimontis	30.55	1700	59.0	141	46.0	1080	11.63	29.5	20.51	36.8	25.55	2	6(3.6)

Table S7. Genbank accession numbers for the gene sequences used to generate the phylogeny from Yu et al. (2018).

Species	12S	16S	CYTB	RAG1	RHOD	TYR
Amolops chunganensis	KX645666.1	KX645666.1	KX645666.1			
Amolops lifanensis	DQ359981.1	DQ204482.1	KJ008458.1		DQ360034.1	DQ360065.1
Amolops mantzorum	DQ359970.1		KJ008405.1	EF088240.1	DQ360023.1	DQ360054.1
Babina pleuraden	JN541324.1	JQ621943.1	KR264150.1	KR264384.1	DQ360011.1	DQ360042.1
Bombina maxima	DQ925758.1	DQ925780.1	EU531274.1			
Brachytarsophrys chuannanensis		KM504261.1				
Bufo andrewsi	AF160764.1	AF160782.1		DQ158353.1	DQ283905.1	
Bufo gargarizans	NC_008410.1	NC_008410.1	JN647482.1	KF666177.1		
Bufo melanostictus	AY458592.1	AB167927.1	AF249082.1	KT031693.1	AF249097.1	
Bufo minshanicus	KM587710.1	KM587710.1				
Bufo tibetanus	AF160766.1	AF160784.1	AF171193.1			
Feirana quadranus	GQ225906.1	GQ225932.1	KX021999.1	HM163591.1	EU979886.1	EU979981.1
Fejervarya multistriata		AF206466.1	AB296096.1	AB526660.1	DQ458271.1	EU980027.1
Hyla annectans chuanxiensis	KP742566.1		FJ226919.1			
Hyla annectans jingdongensis	KP742564.1		AY843821.1		AY844574.1	AY844045.1
Hyla tsinlingensis	KP742646.1	KP212702.1	JX870448.1			
Hylarana guentheri		KF185060.1	KR264131.1	KR264365.1	DQ284009.1	KR264440.1
Kaloula rugifera	JX678894.1	JX678911.1	KT878719.1			
Kaloula verrucosa	KC822507.1	KC822507.1				
Microhyla ornata	AB201177.1	DQ512876.1	AB201223.1	AY364198.1	AY364383.1	KC180221.1
Nanorana parkeri		DQ118498.1	KJ434188.1	HM163584.1	EU979872.1	DQ458276.1
Nanorana ventripunctata	DQ118457.1	EU979839.1		HM163585.1	EU979866.1	EU979959.1
Odorrana grahami	EF453731.1	EU861555.1		EF088257.1	DQ360016.1	DQ360047.1
Odorrana margaretae	DQ359964.1	EU861566.1	KJ815050.1	EF088261.1	DQ360017.1	DQ360048.1
Odorrana nanjiangensis	KF185006.1	?KF185042.1				
Paa boulengeri	EU979791.1	EU979851.1	JX676597.1	HM163604.1	EU979918.1	EU980033.1
Paa yunnanensis	GQ225869.1	GQ225873.1	KF199150.1	HM163593.1	DQ458263.1	EU979976.1
Pelophylax hubeiensis	AF205547.1	AF315137.1				
Pelophylax nigromaculata	DQ359961.1	JQ621942.1	DQ006266.1	AB360184.1	DQ283838.1	DQ360045.1
Polypedates megacephalus	KU840483.1	AY880519.1	AB451722.1	EU924517.1	EU924545.1	KC180271.1
Rana chaochiaoensis	DQ359975.1	DQ289107.1		KX269557.1	DQ360028.1	DQ360059.1
Rana chensinensis	DQ289095.1	DQ289121.1	KM211950.1	KX269551.1		KX269779.1
Rana kukunoris	KX269185.1	KX269185.1	JX486345.1	GQ285780.1	GQ285798.1	GQ285816.1
Rana omeimontis	KX269193.1	DQ289108.1	AF274928.1	KX269558.1		KX269785.1
Rhacophorus chenfui	GQ204763.1	KU840563.1	EU924603.1	EU924519.1	EU924547.1	KU840751.1
Rhacophorus dennysi	DQ019592.1	DQ019609.1	EU924604.1	DQ019512.1	EU215575.1	EU924576.1
Rhacophorus dugritei	EF564471.1	EF564541.1	EU924605.1	GQ285768.1	EU215571.1	EU215601.1
Rhacophorus omeimontis	KU840492.1	KU840564.1	EU924612.1	EU924528.1	EU215565.1	KU840753.1

Figure S1: Directed acyclic graphs representing 20 candidate models that were compared to disentangle the relationships between spawning-site density and altitude, latitude, SVL, age at sexual maturity and longevity in anurans using phylogenetic confirmatory path analyses.

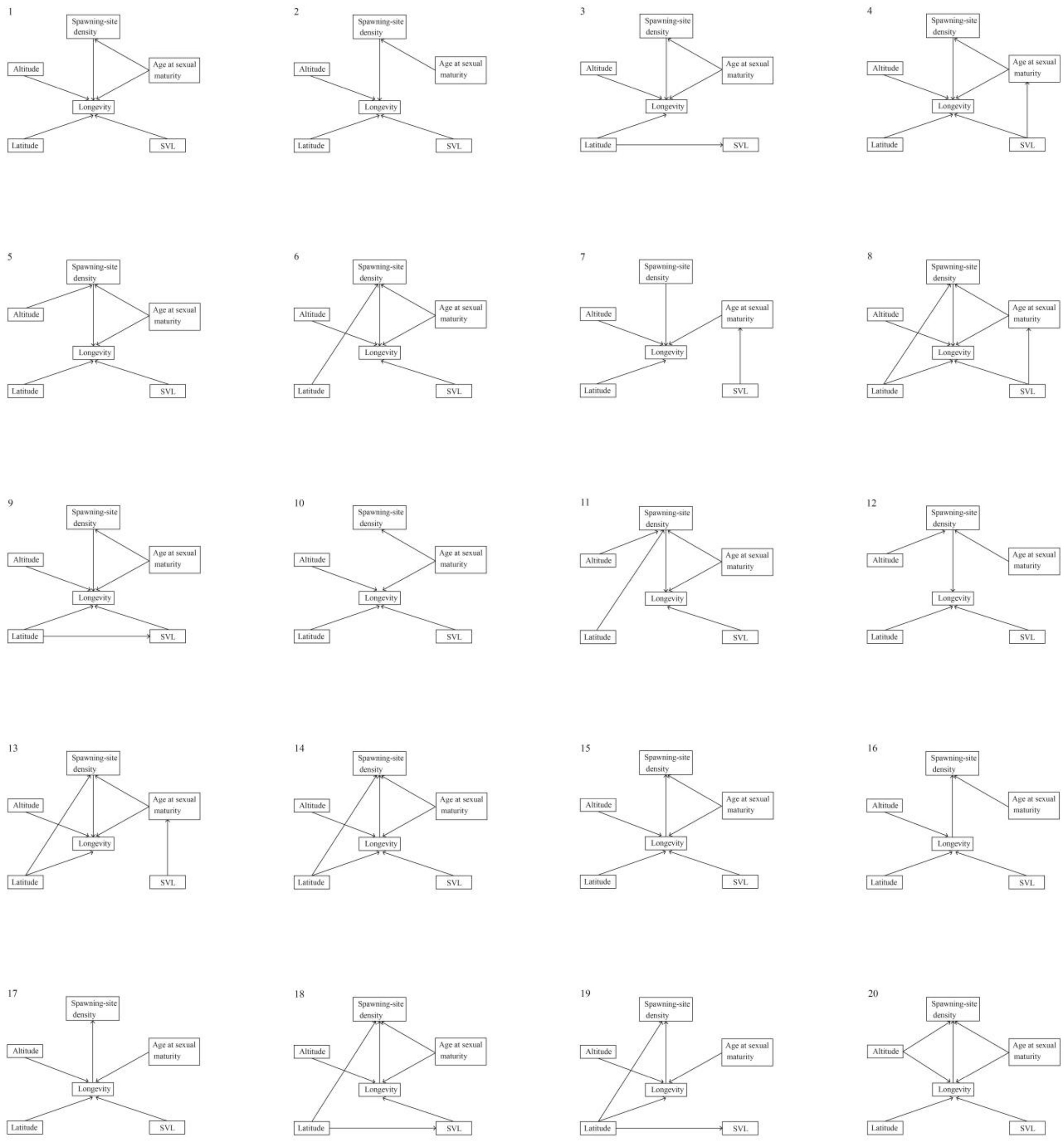

Figure S2: Directed acyclic graphs representing 20 candidate models that were compared to disentangle the relationships between spawning-site group size and altitude, latitude, SVL, age at sexual maturity and longevity in anurans using phylogenetic confirmatory path analyses.

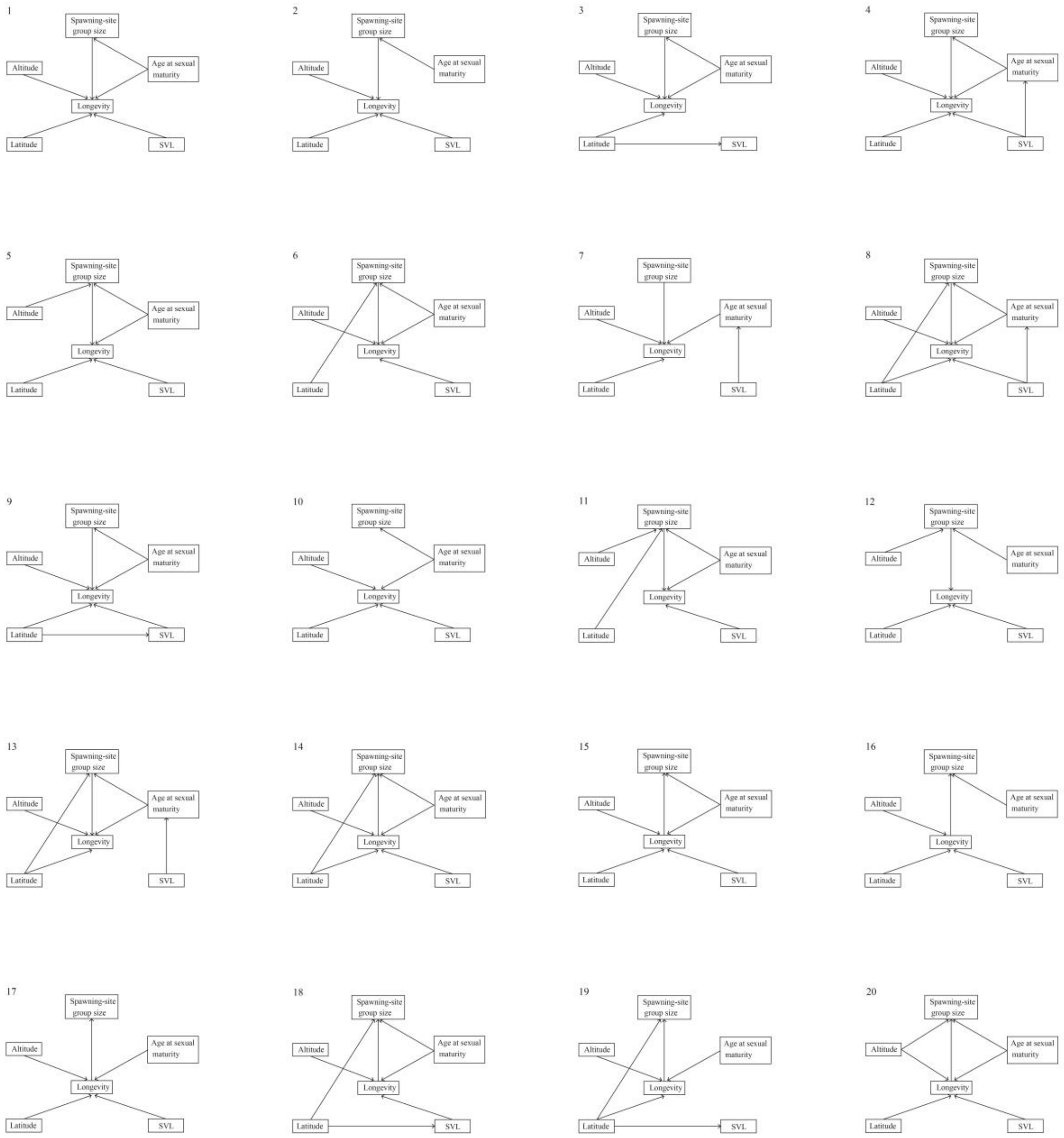

Figure S3: The phylogenetic tree of the 38 species of anurans used in the comparative analysis.

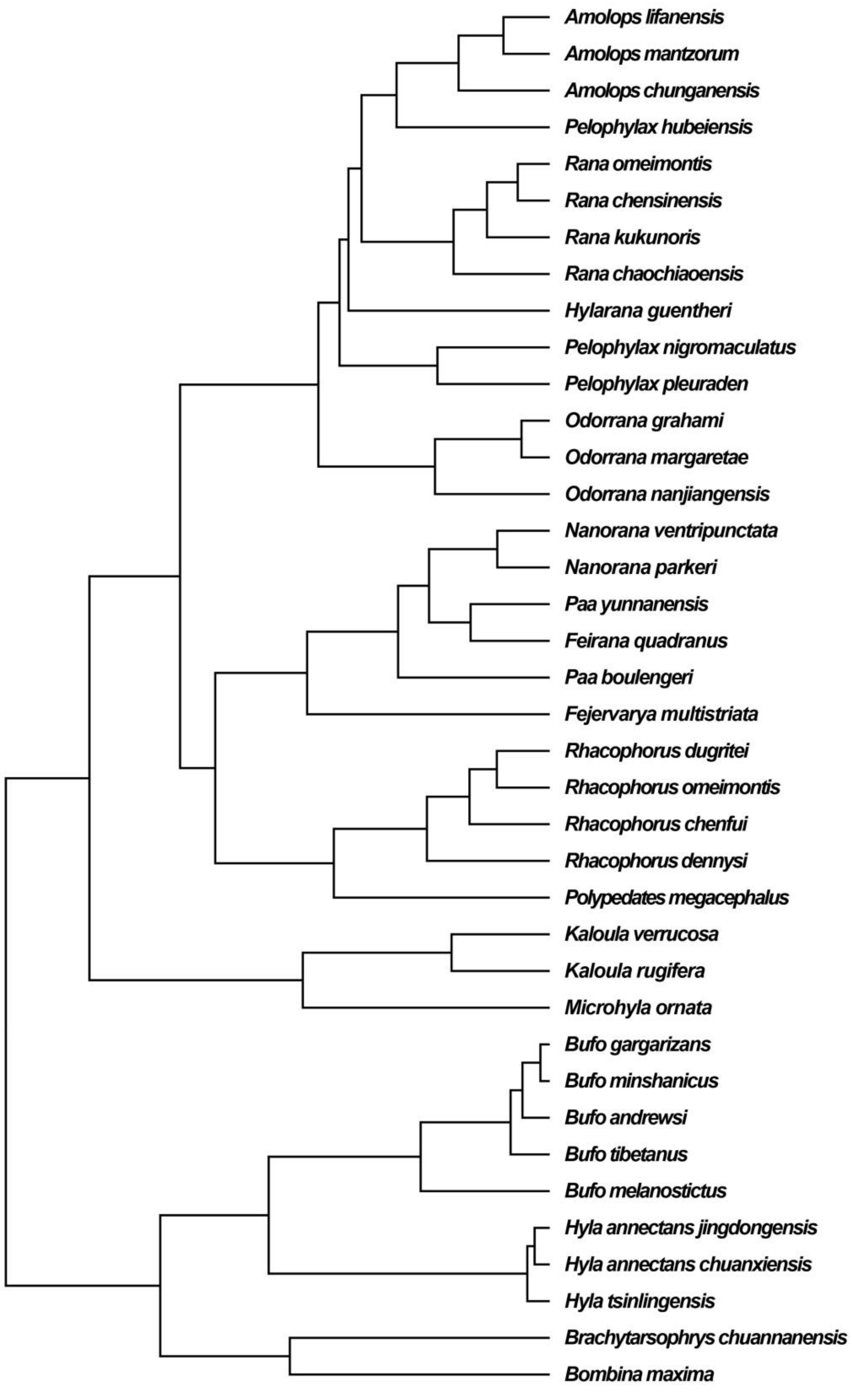

Supplement: Supplementary file 1 — Supplementary [file 41598_2019_50368_MOESM1_ESM.pdf]
